# DRESS syndrome and tuberculosis: Implementation of a desensitization and re-desensitization protocol to recover antituberculosis drugs in a case series at a specialized TB Unit in Lima, Peru

**DOI:** 10.1097/MD.0000000000039365

**Published:** 2024-09-27

**Authors:** Cristian Morán-Mariños, Felix Llanos-Tejada, Juan Salas-Lopez, Renzo Villanueva-Villegas, Antonella Chavez-Huamani, María Vidal-Ruiz, Aaron Rodriguez-Calienes, Renato Casanova-Mendoza

**Affiliations:** a Unidad Especialidad en Tuberculosis, Servicio de Neumología, Hospital Nacional Dos de Mayo, Lima, Peru; b Unidad de Investigación en Bibliometría, Vicerrectorado de Investigación, Universidad San Ignacio de Loyola, Lima, Peru; c Instituto de Investigaciones en Ciencias Biomédicas - INICIB, Facultad de Medicina, Universidad Ricardo Palma, Lima, Peru; d Escuela Profesional de Medicina Humana, Universidad Privada San Juan Bautista, Lima, Peru; e Neuroscience, Clinical Effectiveness and Public Health Research Group, Universidad Científica del Sur, Lima, Peru.

**Keywords:** antituberculosis medication, desensitization, drug hypersensitivity syndrome, tuberculosis

## Abstract

**Rationale:**

Antituberculosis drugs (ATDs) could cause severe and rare reactions, such as Drug Reaction with Eosinophilia and Systemic Symptoms (DRESS) syndrome. Recovering ATDs might guarantee a higher cure rate for tuberculosis patients. Our aim was to evaluate the results of desensitization and re-desensitization to recover ATDs in a case series of patients with DRESS syndrome.

**Patient concerns and diagnoses:**

A retrospective case series study was conducted on patients with DRESS syndrome due to therapy with ATDs from 2021 to 2023. Desensitization and re-desensitization protocols, designed with an algorithm proposed by the Tuberculosis Specialized Unit of the Dos de Mayo National Hospital in Lima, Peru, were implemented.

**Interventions and outcomes:**

A total of 18 patients underwent desensitization or re-desensitization protocols, achieving an overall success rate of 72.2%. The average time for the development of DRESS syndrome due to ATDs was 19 days. Rifampicin (84.2%), isoniazid (68.4%), and pyrazinamide (26.3%) were identified as the main drugs responsible for this adverse reaction. All patients presented with fever and skin rash, with an average eosinophil percentage of 16.7% (interquartile range: 4.5–28.8). Organ involvement (liver, kidney, and heart) was observed in 8 patients, but only 2 patients experienced severe complications due to DRESS syndrome. A significant association was found between the number of ATDs used and eosinophil levels (*P* =.03).

**Lessons:**

The study introduced a desensitization and re-desensitization algorithm for the treatment of DRESS syndrome, notable for its safety, adaptability, and high success rate. This advancement provided healthcare professionals with safer and more effective therapeutic approaches for managing this complex condition.

## 1. Introduction

Drug Reaction with Eosinophilia and Systemic Symptoms (DRESS) syndrome is a rare adverse event due to patient susceptibility to certain medications. The estimated incidence of DRESS syndrome ranges between 1:1000 and 1:10,000 drug exposures and the associated mortality rate may be as high as 20%.^[1,2]^ This reaction is characterized by skin rash, fever, and hematological abnormalities; organ involvement may occur in 69% of cases.^[3]^

Anticonvulsants, allopurinol, antibiotics (sulfonamides, β-lactams, and aminoglycosides), and antivirals are the most common drugs causing DRESS syndrome.^[4,5]^ The reported latency of this syndrome is typically between 2 and 8 weeks after the first exposure to the drug; however, it can present up to 3 months and even after re-exposure.^[3,6]^

Antituberculosis drugs (ATDs) are a rare cause of DRESS syndrome. The first-line ATDs (isoniazid [INH], rifampicin [RIF], ethambutol, and pyrazinamide) are the main cause.^[7]^ Recent years have witnessed increased reports of DRESS syndrome caused by ATDs.^[8]^ This represents an important public health problem since the withdrawal of first-line drugs would reduce the treatment efficacy, increase the treatment cost, prolong treatment, increase the risk of drug resistance, and affect adherence.^[9–11]^ Consequently, desensitization is a fundamental approach to allow the reintroduction of the ATDs which triggered the state of hypersensitivity.

Currently, the proposed desensitization protocols have uncertain results and variable success rates.^[12–14]^ Likewise, the “re-desensitization” method is a new approach, and there is a paucity of empirical data comparing the different regimens for the reintroduction of ATDs.^[15]^

Therefore, the objective of this study was to present an algorithm for desensitization and re-desensitization protocols, as well as to evaluate the results achieved in patients with tuberculosis (TB) with DRESS syndrome in our setting.

## 2. Materials and methods

### 2.1. Study population

This was an observational case series study conducted at the Dos de Mayo National Hospital, in Lima, Peru. From 2021 to 2023, medical records of hospitalized patients with a diagnosis of tuberculosis (sputum confirmation, culture, or genotypic test) and who developed DRESS syndrome due to any ATDs were reviewed. DRESS was defined based on the RegiSCAR-Group Score.^[16]^ The criteria were: score < 2: no case; 2 to 3: possible case; 4 to 5: probable case; >5: definitive case.

### 2.2. Data extraction and analysis

A registry was created based on the previous literature and the information available in the medical records. The following data were retrieved: (a) demographic (age and sex); (b) clinical and laboratory (antecedent, fever, adenopathy, extension and characteristics of skin lesions, involvement of other organs, complications, serology, biopsy, and total and differential leukocyte counts); (c) medication-related characteristics (sensitivity test, drug causing DRESS syndrome; type of desensitization; recovery from desensitization or re-desensitization; final treatment schedule; and length of hospitalization).

Categorical variables were presented as frequency (percentage). Continuous variables were presented as mean ± standard deviation after determining the normality of distribution using the Shapiro–Wilk test. To establish significant differences between the number of medications and the levels of serum eosinophils, we used the Welch’s ANOVA test, which is designed for heterogeneous variances. Statistical analyses were conducted using Stata 17 software.

**Figure 1.**
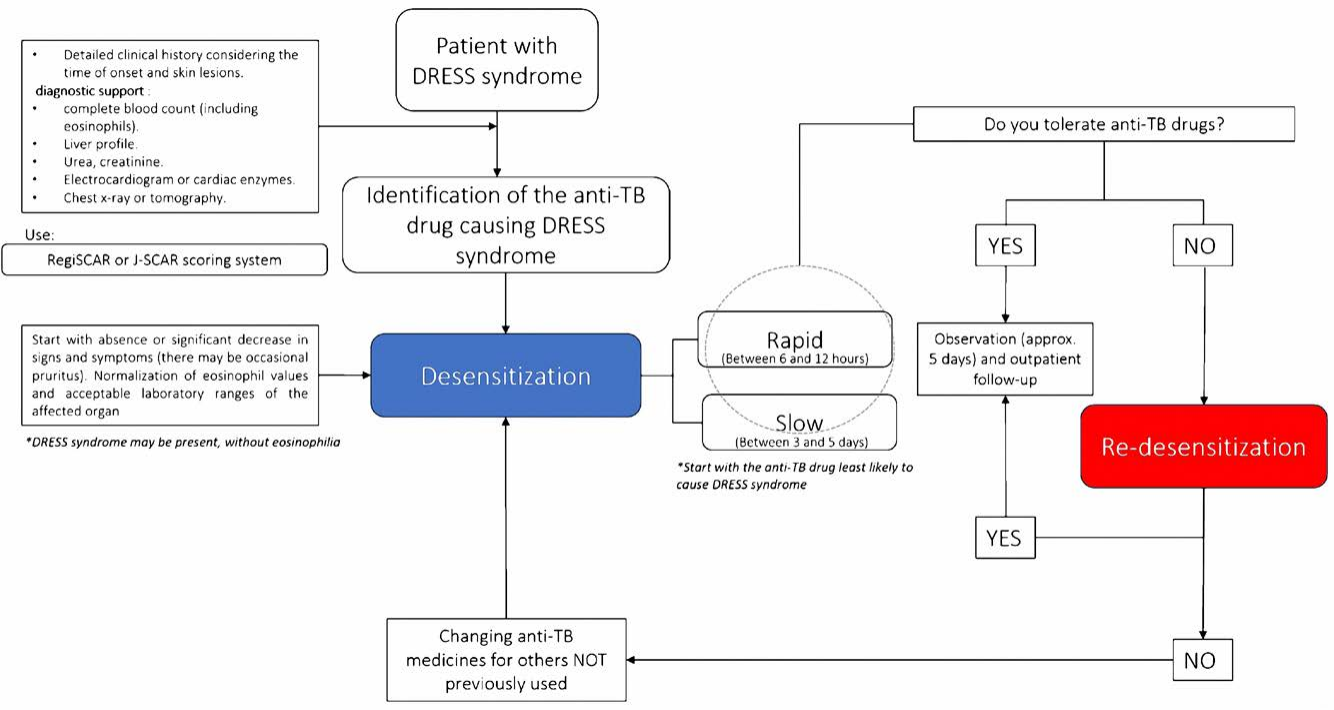
Schematic illustration of the desensitization and re-desensitization algorithms.

Figure 1 illustrates the algorithm for desensitization and re-desensitization in the study population. Table S1, Supplemental Digital Content, http://links.lww.com/MD/N542 provides details on the rapid desensitization technique and its monitoring. Meanwhile, Table S2, Supplemental Digital Content, http://links.lww.com/MD/N543 describes the re-desensitization process for patients whose rapid desensitization failed to achieve successful outcomes with any ATDs.

## 3. Results

The epidemiological, clinical, and laboratory characteristics along with the RegiSCAR scores were presented in Table 1. Out of the 19 patients, 1 patient (no. 11) was lost to follow-up, and sufficient data on the drug that caused DRESS were not available. Cases 6 and 13 developed severe complications due to DRESS syndrome, leading the research group to decide against applying re-desensitization. The remaining cases (no. 3, no. 4, and no. 8) experienced complications related to TB.

**Table 1:** Overview of epidemiological, clinical, and pharmacological characteristics in patients with DRESS syndrome.

Case	Age (year)	Gender	Antecedent/drug susceptibility testing	Interval between anti-TB and development of DRESS syndrome (days)	Anti-TB that cause DRESS syndrome?	Was desensitization or re-desensitization performed?	Were drugs recovered?	Final treatment at hospital discharge*	Hospitalization time (days)	Characteristics of skin lesions	Location of the skin lesion	Organ dysfunction	Values (hemoglobin, leukocytes, lymphocytes, and eosinophils)	RegiSCAR scoring system	Complications
1	28	Male	No/GenoType test (sensitivity to: isoniazid and rifampin)	30	INH, RIF, PZA	Slow desensitization: -INH -PZA	Yes	RIF, LFX, EMB, CS	65	Purpuric and pruriginous erythematous skin rash	Trunk and limbs	Dermic–hepatic	Hemoglobin: 15.2 g/dL Leukocytes: 6701 mm^3^ Lymphocytes: 1121 mm^3^ Eosinophils: 2372 mm^3^ (34.7%)	7 points	No
2	23	Female	DRESS syndrome due to first-line anti-TB drugs/GenoType test (sensitivity to: isoniazid and rifampin)	15	INH, RIF, MFX, LZD	Slow desensitization: INH, RIF, MFX and LZD (failure) Re-desensitization: LZD, MFX	Yes	MFX, CS, CFZ, LZD	92	Maculopapular rash	Diffuse	Dermic	Hemoglobin: 8.5 g/dL Leukocytes: 23200 mm^3^ Lymphocytes: 3254 mm^3^ (28.8%) Eosinophils: 2523 mm^3^ Ig E: 6000 UI/mL	7 points	No
3	30	Male	Hypothyroidism/GenXpert test (sensitivity to: rifampin)	21	RIF	Rapid desensitization: -RIF	Yes	INH, RIF, EMB, PZA	42	Skin xerosis and rash	Trunk and limbs	Dermic–hepatic–renal	Hemoglobin: 11.5 g/dL Leukocytes: 7650 mm^3^ Lymphocytes: 1360 mm^3^ (17.9%) Eosinophils: 693 mm^3^ (6.1%)	7 point	Complications of tuberculosis: Bilateral Pneumothorax and Adrenal Insufficiency
4	22	Male	No/GenXpert test (sensitivity to: rifampin)	26	RIF	Rapid desensitization: - INH - RIF	Yes	INH, EMB, PZA LFX	35	Itchy skin rash and papules	Limbs	Dermic–hepatic	Hemoglobin: 11.7 g/dL Leukocytes: 9270 mm^3^ Lymphocytes: 1329 (11%) Eosinophils: 1636 (17.8%) Ig E: 2271 UI/mL	8 points	Complications of tuberculosis: Left hydropneumothorax
5	31	Male	TB, 10 years ago/GenoType test (sensitivity to: isoniazid and rifampin)	5	INH, RIF	Rapid desensitization: - INH and RIF (failure) Re-desensitization: - RIF	Yes	LFX, RIF, CS, EMB	25	Jaundice, maculopapular rash	Diffuse	Dermic–hepatic	Hemoglobin: 15.7 g/dL Leukocytes: 5990 mm^3^ Eosinophils: 928 mm^3^ (15.4%) Ig E: 1985 UI/mL	7 points	No
6	15	Female	No/GenoType test (sensitivity to: isoniazid and rifampin)	12	INH, RIF, EMB, PZA	Rapid desensitization: - INH - RIF - EMB - PZA	No	No scheme	50	Severe, maculopapular, pruritic rash and desquamation	Diffuse	Dermic–hepatic–renal–cardiac	Hemoglobin: 11.3 g/dL Leukocytes: 13963 mm^3^ Eosinophils: 1619 mm^3^ (11.6%) Lymphocytes: 5282 (37.8%)	8 points	Complications of DRESS syndrome: Multiple organ failure, ICU admission
7	46	Female	No/Löwenstein–Jensen medium (sensitive to first-line drugs)	15	INH, RIF	Rapid desensitization: - INH - RIF	No	AMK, LFX, LZD, CS	30	Maculopapular rash with desquamation	Diffuse	Dermic	Hemoglobin: 11.5 g/dL Leukocytes 8980 mm^3^ Lymphocytes: 2174 mm^3^ (25.1%) Eosinophils: 2217 mm^3^ (25.6%)	7 points	No
8	17	Female	No/GenXpert test (sensitivity to: rifampin)	31	INH, RIF	Slow Desensibilizacion: - INH - RIF	No	Patient died	26	Generalized maculopapular rash and desquamation	Diffuse	Dermic–hepatic	Hemoglobin: 9.7 g/dL Leukocytes: 19150 mm^3^ Lymphocytes: 5783 mm^3^ (30.2%). Eosinophils: 4711 mm^3^ (24.6%)	8 points	Died from complications of TB
9	48	Female	Depression; Alcohol withdrawal/GenoType test (sensitivity to: isoniazid and rifampin)	4	INH, RIF	Rapid desensitization: - INH - RIF	Yes	INH, RIF, EMB, PZA	58	Widespread rash with large scabs and scaling	Diffuse	Dermic	Hemoglobin: 12.5 g/dL Leukocytes: 11740 mm^3^ Lymphocytes: 1197 mm^3^ Eosinophils: 1647 mm^3^ (10.5%)	5 points	No
10	31	Male	AIDS (2004) with ART/GenoType test (sensitivity to: isoniazid and rifampin)	35	INH, RIF, EMB	Rapid desensitization: - INH - RIF - MEB	Yes	AMK, LFX, CS, PZA	27	Maculopapular rash with desquamation	Trunk	Dermic	Hemoglobin: 15.6 g/dL Leukocytes (9470 mm^3^ Lymphocytes: 1136 mm^3^ (12%) Eosinophils: 1705 mm^3^ (18%)	8 points	No
11	19	Male	No/GenXpert test (sensitivity to: rifampin)	12	NA	NA	NA	Discharge without treatment	13	Maculopapular rash with desquamation	Trunk and upper limbs	Dermic	Hemoglobin: 13.3 g/dL Leukocytes: 10,519 mm^3^ Lymphocytes: 3406 mm^3^ (32.3%) Eosinophils: 1654 mm^3^ (15.7%)	6 points	No
12	24	Male	TB MDR/GenoType test (resistant to: isoniazid and rifampin)	28	LZD, PZA	Slow desensitization: - LZD and PZA (failure) Re-desensitization: - LZD	Yes	LZD, BDQ, AMK, CS, CFZ, ETO	70	Itchy skin rash and papules	Diffuse	Dermic–hepatic	Hemoglobin: 9.3 g/dL Leukocytes: 13,235 mm^3^ Lymphocytes: 840 Eosinophils: 2276 (17.2%)	7 points	No
13	29	Male	No/Löwenstein–Jensen medium (sensitive to first-line drugs)	15	INH, RIF, EMB, PZA	Slow desensitization: - INH - RIF - EMB - PZA	No	No scheme	35	Severe, maculopapular, pruritic rash and desquamation	Diffuse	Dermic–hepatic–renal–cardiac	Hemoglobin: 12.1 g/dL Leukocytes: 10,650 mm^3^ Lymphocytes: 2032 mm^3^ (32.3%) Eosinophils: 2884 mm^3^ (27.1%)	8 points	Complications of DRESS syndrome: Hemodialysis
14	42	Male	Alcohol consumption/GenoType test (sensitivity to: isoniazid and rifampin)	18	RIF, PZA	Slow desensitization: - RIF - PZA (failure) Re-desensitization: PZA (failure)	Yes	RIF, INH, EMB	30	Maculopapular rash with desquamation	Trunk and upper limbs	Dermic	Hemoglobin: 10.3 g/dL Leukocytes: 11,234 mm^3^ Lymphocytes: 1360 mm^3^ (17.9%) Eosinophils: 893 mm^3^ (8.3%)	7 points	No
15	33	Female	No/GenXpert test (sensitivity to: rifampin)	8	INH, RIF	Rapid desensitization: - INH (failure) - RIF	Yes	LFX, RIF, CS, EMB	35	Maculopapular rash with desquamation	Diffuse	Dermic	Hemoglobin: 11.7 g/dL Leukocytes: 7769 mm^3^ Eosinophils: 1756 mm^3^ (20.1%)	7 points	No
16	45	Male	TB, 15 years ago/GenXpert test (sensitivity to: rifampin)	25	INH	Rapid desensitization: - INH	No	RIF, LFX, EMB, PZA	28	Skin xerosis and rash	Diffuse	Dermic	Hemoglobin: 10.5 g/dL Leukocytes: 11,320 mm^3^ Lymphocytes: 2231 mm^3^ Eosinophils: 538 mm^3^ (4.7%)	6 points	No
17	22	Female	No/Löwenstein–Jensen medium (sensitive to first-line drugs)	28	INH, RIF	Rapid desensitization: - RIF and INH (failure) Re-desensitization: - INH - RIF (failure)	Yes	LFX, INH, LZD, CFZ, CS, PZA	32	Maculopapular rash with desquamation	Diffuse	Dermic	Hemoglobin: 11.9 g/dL Leukocytes: 9334 mm^3^ Lymphocytes: 753 mm^3^ (14.9%) Eosinophils: 1227 mm^3^ (13.4%)	7 points	No
18	35	Female	Depression/GenXpert test (sensitivity to: rifampin)	14	INH, RIF	Slow desensitization: - RIF (failure) - INH Re-desensitization: -RIF	Yes	RIF, INH, EMB, PZA	35	Maculopapular rash with desquamation	Trunk and upper limbs	Dermic	Hemoglobin: 9.5 g/dL Leukocytes: 8853 mm^3^ Eosinophils: 1911 mm^3^ (21.1%)	8 points	No
19	32	Male	Consumption of illicit substances/GenoType test (sensitivity to: isoniazid and rifampin)	22	RIF	Rapid desensitization: - RIF	Yes	RIF, INH, EMB, PZA	24	Maculopapular rash	Trunk	Dermic	Hemoglobin: 11 g/dL Leukocytes: 10,221 mm^3^ Lymphocytes: 1233 mm^3^ Eosinophils: 1249 mm^3^ (13.2%)	6 points	No

* Anti-TB drugs: isoniazid (INH), rifampicin (RIF), ethambutol (EMB), pyrazinamide (PZA), cycloserin (CS), clofazimine (CFZ), ethionamide (ETO), amikacin (AMK), levofloxacin (LFX), linezolid (LZD), moxifloxacin (MFX), bedaquiline (BDQ).

Among the 19 patients diagnosed with DRESS syndrome, 57.8% were male. The average age was 30.1 ± 9.7 years. It took a median of 19.1 days (interquartile range [IQR]: 4–35) from the onset of antituberculosis treatment to the development of DRESS syndrome. We administered the desensitization protocol to all 18 patients who were part of this study: 7 received rapid desensitization, achieving a success rate of 63.6%, while only 2 of the patients (28.5%) successfully underwent slow desensitization. Additionally, six patients who initially did not benefit from the desensitization efforts were subjected to a re-desensitization process, through which we were able to successfully reintroduce one or more ATDs for each of them, achieving a 100% success rate (Table 2).

**Table 2:** Summary of epidemiological characteristics, anti-TB, and DRESS syndrome.

		N	%
Age (year)		15–483	30.1*; 9.7†
Gender			
Male		11	57.8
Female		8	42.1
Interval between anti-TB and DRESS (Days)		4–35	19.1*; sd: 9.1†
Were drugs recovered (n = 18)?			
Yes		13	72.2%
No		5	27.7%
Success rate (desensitization and re-desensitization)			
Only desensitization:			
Rapid (n = 11)	success	7	63.6%
Slow (n = 7)	success	3	42.9%
Re-desensitization (n = 6)§	success	5	83.3%
Drugs that caused DRESS Syndrome?			
One		4	22.2%
Two		8	44.4%
Three or more		6	33.3%
Hospital time		13–92‡	35*; 19.3†

* Mean.

† Standard deviation.

‡ Interquartile range.

§ Re-desensitization was implemented for some patients who did not respond favorably to the initial desensitization process. Recovery of at least 1 DAT was considered a success.

Table 3 outlines the clinical and laboratory features observed. All patients in our case series exhibited fever and rash, with the resolution of symptoms occurring in 15 days or more. Seventeen of the 19 patients exhibited eosinophilia (mean percentage of eosinophils: 16.7%, IQR: 4.5–28.8). Lymphadenopathy and atypical lymphocytes were observed in the lowest percentage of cases (<45%). The liver was the most affected organ, involved in 62.5% of cases.

**Table 3:** Clinical and laboratory profile of patients with TB and DRESS syndrome.

	N	%
*Diagnostic criteria (n = 19*)		
Fever	19	100%
Lymphadenopathy	8	42.1%
*Skin rash*	19	100%
*At least two of: edema, infiltration, purpura, scaling*	19	100%
*Resolution in > 15 days*	19	100%
*Internal organ involved*	8	42.1%
*Eosinophilia*	17	89.4%
*Atypical lymphocytes*	7	36.8%
*Organs affected (n = 8*)		
*An organ*	5	62.5%
Hepatic		
*Multiorganic*	*3*	*37.5%*
hepatic–renal		
hepatic–renal–cardiac		
*Laboratory values (n = 19*)	Interquartile range (IQR)	Mean/standard deviation
Hemoglobin (g/dL)	8.5–15.7	11.7/2.05
Leukocytes (mm^3^)	5900–23,200	11,008/4174
Lymphocytes (mm^3^)	753–5783	2155/1532
Eosinophils (mm^3^)	538–4711	1816/945
Eosinophils (%)	4.5–28.8%	16.7/6.5

**Figure 2.**
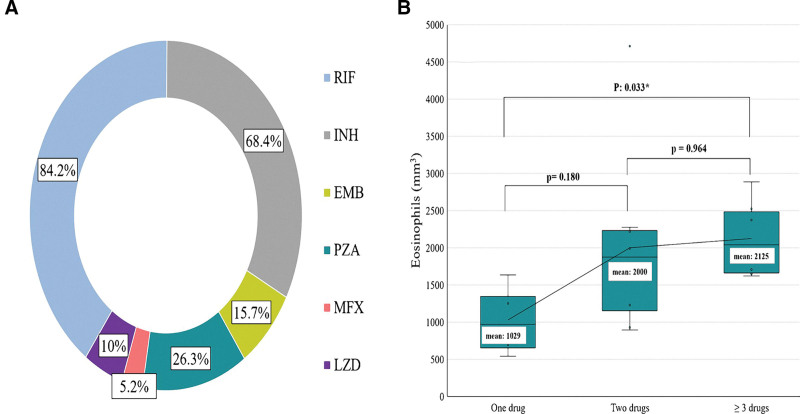
Frequency of drugs causing DRESS syndrome, and the relationship between the number of drugs and eosinophil count.

Rifampicin was identified as the causative drug in 84.2% of cases, followed by isoniazid at 68.4% and pyrazinamide at 26.3%. Two patients (10%) developed DRESS syndrome due to linezolid, while only 1 patient (5%) was affected by moxifloxacin (Fig. 2a). In the ANOVA analysis, we observed a statistically significant difference in means (*P* = .033) between the absolute eosinophil levels and the number of DATs involved in DRESS syndrome. Specifically, there was a mean difference of 1.096 mm³ between the group treated with a single DAT and those treated with three or more DATs (Fig. 2b).

**Figure 3.**
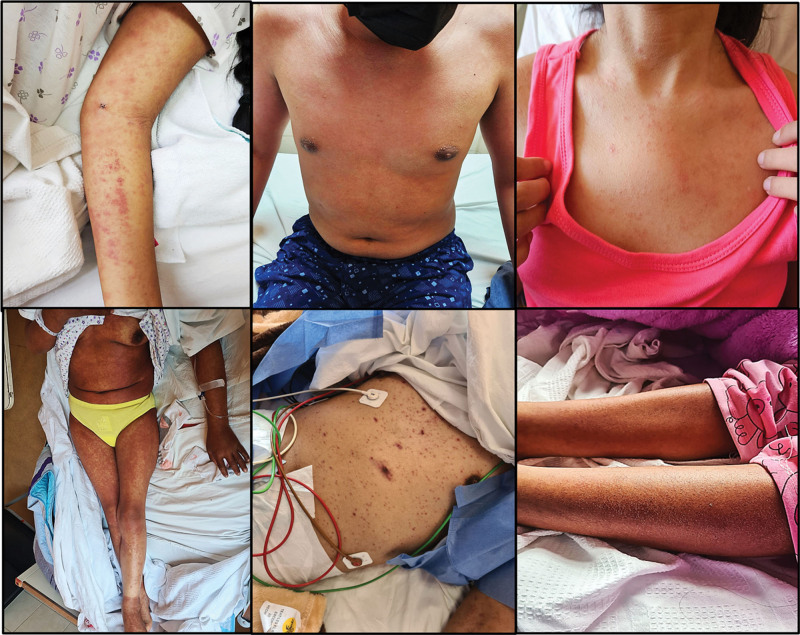
Patients with tuberculosis and DRESS syndrome in different dermatological stages.

Figure 3 shows representative patients with different skin manifestations of the DRESS syndrome. The most common pattern was diffuse maculopapular rash predominantly involving the trunk.

## 4. Discussion

This retrospective study revealed that desensitization is rarely applied in our setting despite several successful cases reported in the literature.^[12,13,17]^ Re-desensitization is a new method that offers favorable prospects of restarting the treatment with the culprit ATDs. This is backed up by the overall success rate of 72.2% observed in our case series. Of note, the patients who underwent slow desensitization did not show a favorable response; however, we believe that by applying re-desensitization these drugs would have been recovered. Buhuari et al^[18]^ obtained favorable results (80%) after readministering the drug. There is a paucity of published desensitization and re-desensitization protocols that have been tested in a large sample. Even though slow desensitization was unsuccessful in our cohort, studies published in the East such as those by Kobashi et al^[19]^ and Thong et al,^[15]^ have reported success rates exceeding 75%; however, the days to recover the ATDs were very long (up to 14 days). Our rapid desensitization protocol was 63.6% effective, and a similar experience was reported by Matz et al^[20]^ who reported a success rate of 77.8%.^[14]^ Re-desensitization of the drug ensured that our patients did not present any important adverse event, nor did we identify any clinical predictors of mortality. This is supported by previous studies on re-desensitization.^[21,22]^ Therefore, the authors consider it important to propose the first-ever algorithm to recover ATDs (Fig. 1). This is an important aspect as it challenges the current “expert opinion” of not reintroducing the ATDs that trigger DRESS syndrome.

### 4.1. Clinical features and pathophysiological mechanisms

In our study, the sex of the patient did not seem to be an important factor, although a previous study reported a slightly greater predilection for the female sex.^[4]^ In our study, the time elapsed from exposure to ATD to the onset of DRESS syndrome showed a wide range (4–35 days) with a mean of 19.1 days. This is consistent with the typical two-week presentation caused by other drugs.^[2,3]^ In the context of TB, it is more common to find non-immediate reactions, not only due to the number of medications the patient receives but also due to the genetic polymorphism of the ATDs-metabolizing enzymes with the presence of several human leukocyte antigen haplotypes and genetic variants.^[23,24]^ Yu et al^[25]^ determined the allelic frequencies of genes and found that (cytochrome P [CYP]) *CYP2B6*, *CYP2E1*, and *NAT2* genes presented a higher risk of developing adverse drug events. This may be relevant to our study population since the majority of patients who developed DRESS syndrome showed a reaction to 2 or more drugs (77.3%). Likewise, the simultaneous occurrence of allergic and/or hypersensitivity reactions to multiple drugs makes it challenging to identify the responsible drug.^[5]^

### 4.2. ATDs regimen to initiate desensitization

Our results have important implications for the order of medication with which to initiate desensitization. We must start with the least likely ATDs in order to recover as many drugs as possible that did not cause DRESS syndrome. This will generate a lower risk of adverse events and prolonged hospital stay since it would not require one to wait for a significant decrease in symptoms or laboratory indices to attempt re-desensitization. We found that RIF and INH were an important cause in more than 68% of the cases of DRESS syndrome. While starting with quinolones, we would expect a reaction in <10% of the cases. We had two cases of DRESS syndrome due to linezolid (cases 2 and 12), which is extremely rare; this is consistent with the results published by Jin et al^[8]^ who identified RIF (75.5%) and INH (62.3%) as the main cause, while the drugs in group A or B were responsible for <5% cases. Therefore, we propose to start desensitization with first-line drugs under the following regimen: pyrazinamide first, followed by ethambutol, INH, and finally RIF. Starting with any group A drug appears to be very safe and there is not enough evidence to recommend an order. An individual assessment of risks and benefits must be carried out for each patient, and the final decision should be made by the treating physician.^[26,27]^

### 4.3. Do eosinophils contribute to the increase in adverse events in DRESS syndrome triggered by ATDs?

We discovered a significant difference in eosinophil levels when comparing the number of ATDs involved (*P* = .03). Six patients from our study (no. 1, 2, 7, 8, 12, and 13) exhibited eosinophil counts exceeding 2.000 mm³ and presented with involvement of 2 or more organs. Interestingly, a similar pattern of organ involvement was observed in cases where eosinophil counts were below 1.000 mm^3^. This is supported by a previous study of more than 2.234 TB patients in which eosinophilia was identified in 17.8% of the cases, but only 7.2% had cutaneous adverse reactions manifesting only as itch and rash, with none developing organ involvement.^[28]^ Therefore, the authors do not consider that eosinophil count has a predictive value for adverse events, as observed in other lung diseases.^[29,30]^ There is also no clear evidence that patients with human immunodeficiency virus/TB coinfection or patients with fulminant hepatitis who have developed a drug-induced severe skin reaction are at a higher risk of mortality (mortality rates <5% and 9.7%, respectively).^[31,32]^ Therefore, despite the reported mortality rate of 10% to 20%, the percentage largely depends on the causative drug. Certainly, the context of each patient must be understood before starting desensitization, since there are important risk factors that can lead to rapid disease progression, decreasing the survival rate.^[33,34]^ Therefore, we do not recommend desensitization in patients with significant lung injury or significant comorbidities.^[10]^

### 4.4. Research limitations

It is crucial to acknowledge certain limitations inherent to our study that might affect the interpretation and generalizability of its results. Firstly, our study’s retrospective design, conducted at a single center, involved a relatively small sample size. This configuration may introduce biases in the data collection process, constraining our ability to generalize our findings to broader or more diverse populations. Moreover, being based at a single center may not reflect the variability in clinical practices or patient responses to desensitization protocols across different healthcare settings.

Given the limitations of our study, it is crucial to conduct further research, particularly multicenter studies with larger sample sizes. This approach will help capture the variability in clinical practices and patient responses to desensitization protocols across different healthcare settings, enhancing our findings’ generalizability. Comparing the effectiveness of various desensitization protocols (fast vs slow) through collaborative networks could provide stronger evidence to refine patient management strategies. Additionally, prospective studies could address the biases inherent in retrospective analyses, enabling a more precise evaluation of desensitization protocols across diverse patient groups.

Given these considerations, our study’s findings should be interpreted with caution. Although promising, particularly in terms of the success of our desensitization and re-desensitization algorithm, it is imperative for future research to investigate these topics more thoroughly and methodically. Such efforts will enhance our results’ credibility and yield more dependable clinical recommendations for managing complex conditions like DRESS syndrome in TB patients.

## 5. Conclusions

We presented the first case series in Latin America detailing the clinical features of DRESS syndrome due to ATDs. An innovative aspect was the proposal and application of a desensitization and re-desensitization algorithm, which, developed under a protocol emphasizing safety and individual adaptability, achieved a remarkable success rate. This demonstrates its effectiveness in managing this complex syndrome. The implementation of this algorithm represents an important advance in the treatment of DRESS syndrome, offering health professionals more effective and safer therapeutic options.

## Acknowledgments

We extend our sincere gratitude to the Pulmonary Resident Doctors for their crucial role in the desensitization and re-desensitization processes of the patients. Similarly, we appreciate the patients’ collaboration and commitment to the study.

## Author contributions

**Conceptualization:** Cristian Morán-Mariños, Felix Llanos-Tejada.

**Formal analysis:** Cristian Morán-Mariños, Felix Llanos-Tejada.

**Investigation:** Felix Llanos-Tejada, Juan Salas-Lopez, Renzo Villanueva-Villegas, Antonella Chavez-Huamani, María Vidal-Ruiz, Aaron Rodriguez-Calienes, Renato Casanova-Mendoza.

**Methodology:** Cristian Morán-Mariños, María Vidal-Ruiz, Aaron Rodriguez-Calienes, Renato Casanova-Mendoza.

**Supervision:** Cristian Morán-Mariños, Felix Llanos-Tejada, Juan Salas-Lopez, Renzo Villanueva-Villegas, Antonella Chavez-Huamani.

**Validation:** Cristian Morán-Mariños, Juan Salas-Lopez, María Vidal-Ruiz, Aaron Rodriguez-Calienes, Renato Casanova-Mendoza.

**Visualization:** Renzo Villanueva-Villegas.

**Writing – original draft:** Cristian Morán-Mariños, Felix Llanos-Tejada, Juan Salas-Lopez, Renzo Villanueva-Villegas, Antonella Chavez-Huamani, María Vidal-Ruiz, Aaron Rodriguez-Calienes, Renato Casanova-Mendoza.

**Writing – review & editing:** Cristian Morán-Mariños, Felix Llanos-Tejada, Juan Salas-Lopez, Renzo Villanueva-Villegas, Antonella Chavez-Huamani, María Vidal-Ruiz, Aaron Rodriguez-Calienes, Renato Casanova-Mendoza.

## Supplementary Material


