# Supplementary material for: DRESS syndrome and tuberculosis: Implementation of a desensitization and re-desensitization protocol to recover antituberculosis drugs in a case series at a specialized TB Unit in Lima, Peru
Source: Medicine (Baltimore). 2024 Sep 27;103(39):e39365. doi: 10.1097/MD.0000000000039365 (PMC11441958; doi:10.1097/MD.0000000000039365)
Supplement: Supplementary file 1 [file medi-103-e39365-s001.docx]

**DRESS Syndrome and Tuberculosis: Implementation of a Desensitization and Re-desensitization Protocol to Recover Anti-Tuberculosis Drugs in a Case Series at a Specialized TB Unit in Lima, Peru.**

**Journal:** Medicine®

**Rapid desensitization and re-desensitization protocol**

**Table S1** shows the rapid desensitization protocol for the identified first-line drugs (INH, RIF, EMB, PZA) that triggered the DRESS syndrome. The method involves starting the medication that is sought to be recovered in low doses and dosing at intervals of 20 minutes (in the first 3 hours) and 30 minutes (last hour) until the maximum corresponding dose for that medication is reached. Because this method aims to recover the drugs in the shortest possible time, the patient must be strictly monitored for signs and symptoms of jaundice, nausea, vomiting, rash, or other abnormal symptoms. If the patient’s condition is satisfactory, it is appropriate to administer a tablet or capsule every 12 hours on the second day. If tolerated on the 3rd day, the medication can be administered at a dose per kg/weight every 24 hours.

|  | Dose (mg) | | | | Outcome | | | | | | |
| --- | --- | --- | --- | --- | --- | --- | --- | --- | --- | --- | --- |
|  | INH | RIF | EMB | PZA | Jaundice | Nausea | Vomiting | Rash | Other | Observations |  |
| Date: |  |  |  |  |  |  |  |  |  |  |  |
| 09:00 | 0.1 | 0.1 | 0.1 | 0.1 |  |  |  |  |  |  |  |
| 09:20 | 0.5 | 0.5 | 0.5 | 0.5 |  |  |  |  |  |  |  |
| 09:40 | 1 | 1 | 1 | 1 |  |  |  |  |  |  |  |
| 10:00 | 2 | 2 | 2 | 2 |  |  |  |  |  |  |  |
| 10:20 | 4 | 4 | 4 | 4 |  |  |  |  |  |  |  |
| 10:40 | 8 | 8 | 8 | 8 |  |  |  |  |  |  |  |
| 11:00 | 16 | 16 | 16 | 16 |  |  |  |  |  |  |  |
| 11:20 | 32 | 32 | 32 | 32 |  |  |  |  |  |  |  |
| 11:40 | 50 | 50 | 50 | 50 |  |  |  |  |  |  |  |
| 12:00 | 100 | 100 | 100 | 100 |  |  |  |  |  |  |  |
| 12:30 |  | 150 | 200 | 250 |  |  |  |  |  |  |  |
| 13:00 |  | 300 | 400 | 500 |  |  |  |  |  |  |  |

**Table S1.** Rapid desensitization protocol with first-line antituberculosis drugs

If tolerated, it corresponds one tablet / capsule every 12 hours the next day.
If tolerated on the 2nd day, it corresponds to a dose per kg/weight every 24 hours the following day.
