# Supplementary material for: DRESS syndrome and tuberculosis: Implementation of a desensitization and re-desensitization protocol to recover antituberculosis drugs in a case series at a specialized TB Unit in Lima, Peru
Source: Medicine (Baltimore). 2024 Sep 27;103(39):e39365. doi: 10.1097/MD.0000000000039365 (PMC11441958; doi:10.1097/MD.0000000000039365)
Supplement: Supplementary file 2 [file medi-103-e39365-s002.docx]

**DRESS Syndrome and Tuberculosis: Implementation of a Desensitization and Re-desensitization Protocol to Recover Anti-Tuberculosis Drugs in a Case Series at a Specialized TB Unit in Lima, Peru.**

**Journal:** Medicine®

**Rapid desensitization and re-desensitization protocol**

**Table S2** shows the re-desensitization protocol for those patients for whom rapid desensitization failed, and for whom it was necessary to recover some drug if possible. The protocol is carried out over 7 days. The method involves dividing the minimum dose per day (until the second day) and administering at intervals of 12 or 6 hours. As of the third day, the dose can be increased by 50% of the total dose (administered in 3 equal doses); between the fourth and fifth day, the dose is increased almost in its entirety, with the aim of prolonging the dose for greater than 12 hours. Finally, on days 6 and 7 (adjusted dose), the total dose of the drug is administered. It is necessary to carry out periodic laboratory controls on the days mentioned. Finally, a practical way of preparing the doses is mentioned.

**Table S2.** Example of a successful "re-desensitization" protocol with linezolid

| Start day | Time | Dose | Laboratory control^*^ | **Preparation** |
| --- | --- | --- | --- | --- |
| 1 | 8:00 A.M. | 0.1mg | Hemogram and liver profile | Example to prepare 0.1mg of LZD. Dilute a Linezolid tablet in 60cc of water a) 600mg---60cc, therefore: b) 10 mg in 1 cc; we will add 9cc of water c) 10mg --- 10cc of water, therefore: d) 0.1 mg is 0.1 cc (use a 1 cc syringe) |
|  | 20:00 P.M. | 0.5mg |  |  |
| 2 | 08:00 A.M. | 8mg |  |  |
|  | 16:00 P.M. | 16mg |  |  |
| 3 | 8:00 A.M. | 100mg | Hemogram and liver profile |  |
|  | 14:00 P.M. | 100mg |  |  |
|  | 20:00 P.M. | 100mg |  |  |
| 4 | 8:00 A.M. | 200mg |  | To get 8mg of LZD. Dilute a tablet in 60 cc of water a) 600mg --- 60cc, therefore: b) 10mg in 1cc c) 8mg will be 0.8cc (use 1cc syringe) |
|  | 21:00 P.M. | 300mg |  |  |
| 5 | 8:00 A.M. | 200mg | Hemogram and liver profile |  |
|  | 22:00 P.M. | 400mg |  |  |
| 6 | 8:00 A.M. | 600mg |  |  |
| 7 | 8:00 A.M. | 600mg* | Hemogram and liver profile |  |

Adjust to the required dose, in the case of linezolid 10-20 mg/kg/day or only a 600mg dose once a day

*Before starting the medication
